# Intracavernous Internal Carotid Artery Aneurysm Presenting as Acute Diplopia: A Case Report

**DOI:** 10.5811/cpcem.2020.3.45266

**Published:** 2020-07-20

**Authors:** Austin Brown, Heath Jolliff, Douglas Poe, Michael Weinstock

**Affiliations:** * Adena Regional Medical Center, Department of Emergency Medicine, Chillicothe, Ohio; † Wexner Medical Center at The Ohio State University, Department of Emergency Medicine, Columbus, Ohio

**Keywords:** Sixth cranial nerve palsy, diplopia, intracavernous internal carotid artery

## Abstract

**Introduction:**

Diplopia is an uncommon emergency department (ED) complaint representing only 0.1% of visits, but it has a large differential. One cause is a cranial nerve palsy, which may be from a benign or life-threatening process.

**Case Report:**

A 69-year-old female presented to the ED with two days of diplopia and dizziness. The physical exam revealed a sixth cranial nerve palsy isolated to the left eye. Imaging demonstrated an intracavernous internal carotid artery aneurysm. The patient was treated with embolization by neurointerventional radiology.

**Discussion:**

The evaluation of diplopia is initially divided into monocular, usually from a lens problem, or binocular, indicating an extraocular process. Microangiopathic disease is the most common cause of sixth nerve palsy; however, more serious etiologies may be present, such as an intracavernous internal carotid artery aneurysm, as in the patient described. Imaging modalities may include computed tomography or magnetic resonance imaging.

**Conclusion:**

Some causes of sixth nerve palsy are benign, while others will require more urgent attention, such as consideration of an intracavernous internal carotid artery aneurysm.

## INTRODUCTION

Dizziness, imbalance, vertigo, or lightheadedness account for over 4.3 million emergency department (ED) visits annually in the United States;^1^,^2^ vision changes, such as double vision or diplopia, represent 0.1% of ED visits.^3^ The initial differentiation of a “double vision” visit is to determine monocular vs binocular diplopia; this will help focus the urgency of imaging, diagnostic testing and disposition. The most common source of diplopia, accounting for 50% of diagnosis, is from an isolated cranial nerve palsy involving the sixth cranial nerve (abducens nerve).^4^ A diagnosis of sixth cranial nerve palsy can often be referred to neurology in an urgent manner; however, with certain risk factors and associated symptoms further imaging in the ED may be warranted.

One pathology that may cause an isolated sixth cranial nerve palsy is an intracavernous internal carotid artery aneurysm, which is present in only 3% of cases of dizziness.^5^ We present a case where a 69-year-old female presented to the ED with dizziness secondary to diplopia. The only significant finding on physical exam was a left sixth cranial nerve palsy.

## CASE REPORT

A 69-year-old female presented to the ED with two days of dizziness and double vision that was present only when both eyes were open and resolved when she closed one or both eyes. She denied any concomitant symptoms such as headache, neck pain, paresthesia, numbness, or weakness. Approximately five days prior to ED evaluation the patient was diagnosed with otitis media and sinusitis at an urgent care and placed on cephalexin. The patient had no neurological symptoms present at that time.

**Image 1:**
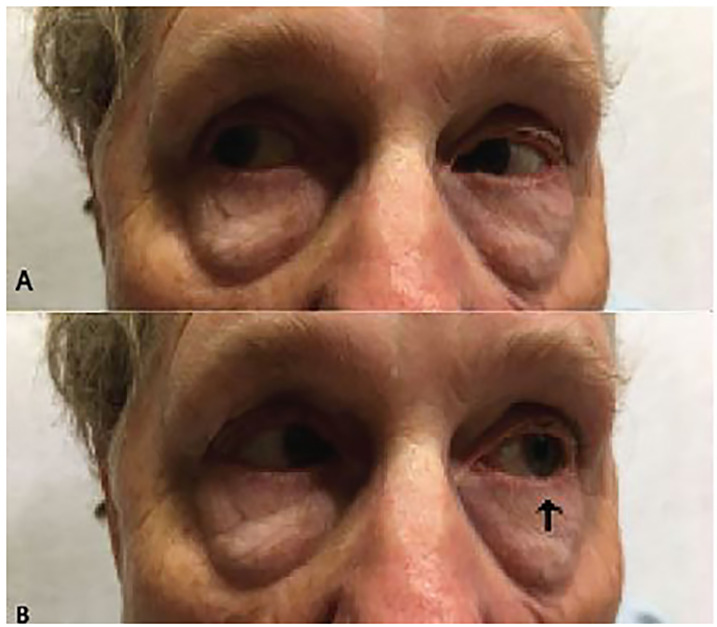
Lateral gaze testing. (A) Right lateral gaze test normal. (B) Left lateral gaze demonstrating left ocular lateral gaze palsy (arrow).

On physical examination the patient was afebrile with pulse of 64 beats per minute, respiratory rate of 16 respirations per minute, blood pressure of 150/83 millimeters of mercury, and oxygen saturation of 96% on room air. The neck, lung, heart, and abdominal exams were normal. Visual acuity was 20/30 in the right eye and 20/25 in the left eye with correction. Ocular exam demonstrated a left lateral gaze palsy of the left eye (Image 1). No other extraocular deficits were identified. The neurologic exam revealed the patient to be alert and oriented to person, place, and time with a Glasgow Coma Scale of 15. No other cranial nerve or neurologic deficits were present. The differential diagnosis included direct sixth nerve compression due to intracranial pathology, intracranial ischemia, carotid artery aneurysm or dissection, and cavernous sinus vasculitis.

**Image 2:**
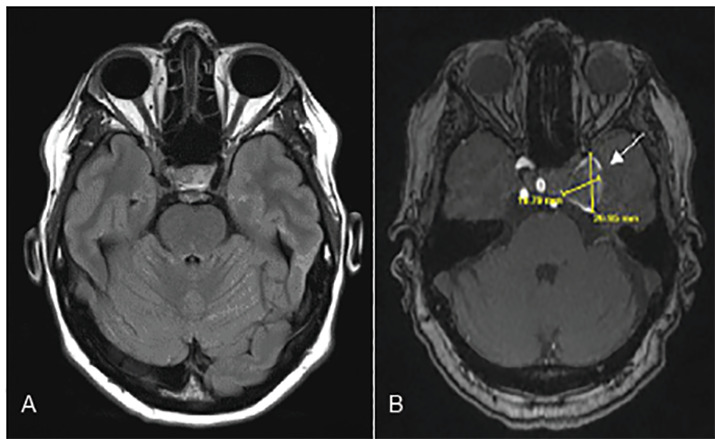
Internal carotid artery aneurysm. (A) Normal brain magnetic resonance imaging (MRI) (image courtesy: Prof. Frank Gaillard, Radiopedia.org) (B) Patient’s MRI showing thrombosed internal carotid artery aneurysm.

A computed tomography (CT) angiography of the head and neck were considered; however, because the patient was allergic to intravenous contrast, we obtained non-contrast magnetic resonance imaging (MRI) of the brain and MR angiography of the brain and neck that demonstrated a large, partially thrombosed left cavernous internal carotid artery aneurysm measuring up to 2.7centimeter (cm) × 2.0cm × 2.1 cm resulting in compression of the sixth cranial nerve (Image 2).

The neurointerventional radiologist was consulted and successfully pipeline embolized the thrombosis. On three-month follow-up the patient’s vision was reported to be intact with resolution of the diplopia and dizziness. The patient continued to have a slight residual left sixth nerve palsy on physical exam.

**CPC-EM Capsule**

What do we already know about this clinical entity?

Sixth cranial nerve palsies are commonly benign but some findings may need further investigation and sometimes involving advanced imaging.

What makes this presentation of disease reportable?

Sixth cranial nerve palsies can be caused by an intracavernous internal carotid artery aneurysm, which needs urgent intervention.

What is the major learning point?

Dizziness is a common emergency department presentation where a full neurological exam including testing all cranial nerves must be done to rule out urgent pathology.

How might this improve emergency medicine practice?

Sixth cranial nerve palsies, although mostly benign, may need advanced imaging where urgent intervention is needed.

## DISCUSSION

The differential for diplopia starts by determining whether the diplopia is monocular, which implicates an intraocular/lens abnormality, or binocular, which indicates an extraocular process. Binocular diplopia results from ocular misalignment, which can be secondary to impaired neuromuscular control of the medial rectus muscle, lateral rectus muscle, or both.^6^ Sixth nerve palsy can be differentiated into six syndromes based on where the nerve travels anatomically (summarized in the table).

Among patients presenting with an eye movement abnormality, a sixth cranial nerve palsy is the most common, representing 50% of cases.^4^ The most common cause of sixth cranial nerve palsy is microangiopathic disease with increased incidence in patients with hypertension and older age^7^; other etiologies include trauma, demyelination and, rarely, neoplasms.^8^ Most causes spontaneously resolve within 2–3 months.^9^ The decision of whether to image the head or neck in the non-traumatic, isolated sixth nerve palsy should be a case-by-case decision. The ophthalmology literature recommends that patients above the age of 50 with risk factors including diabetes or multiple sclerosis may be treated conservatively with management focusing on underlying systemic conditions, and immediate neuroimaging may be delayed.^10^ However, contrast CT or MRI is indicated in patients with other neurological symptoms or signs, patients less than 50 years of age (older patients are more likely to have microangiopathic disease), symptoms that are present for longer than 2–3 months, of if there is diagnostic uncertainty.^11^

The clinical course of an intracavernous carotid aneurysm can be variable and clinical progression can occur; however, symptomatic aneurysms can also improve spontaneously.^11^ Cranial nerve palsies are among the most common complications of intracavernous internal carotid aneurysm.^11^ Cranial nerves that transverse the cavernous sinus include the oculomotor (third cranial nerve), trochlear nerve (fourth cranial nerve), the ophthalmic and maxillary branches of the trigeminal nerve (fifth cranial nerve) and the abducens (sixth cranial nerve).^12^ The diagnosis of an intracavernous carotid aneurysm in an isolated sixth nerve palsy presentation is rare, occurring in up to 3% of cases.^5^

Patients with intracavernous carotid artery aneurysms may be managed with coil embolization, balloon occlusion, or a new technique that involves pipeline diversion.^13^ This novel treatment is now becoming more popular. Three-year follow-up studies have shown that pipeline embolization is safe and effective in the treatment of complex large and giant aneurysms of the intracranial internal carotid artery.^10^

## CONCLUSION

The isolated sixth nerve palsy although normally benign can be caused by an emergent pathology such as an intracavernous internal carotid artery aneurysm as presented in this case. When a patient with diplopia presents to the ED, the physician must use careful history-taking and physical exam skills to find even the most subtle findings to better diagnose and possibly treat a life-threatening pathology.

## Figures and Tables

**Table:** Sixth nerve palsy differentiation.^5^

	Sixth Nerve Palsy Syndromes	Description
1.	Brain stem syndrome	Compressive, ischemic, inflammatory or degeneration within the brain stem
2.	Elevated intracranial pressure syndrome	Increased pressures in subarachnoid space caused by hemorrhage, infections, or infiltrates
3.	Petrous apex syndrome	Compression under petroclinoid ligament
4.	Cavernous sinus syndrome	Pathologies involving the cavernous sinus include nasopharyngeal carcinoma, intracavernous internal carotid aneurysm, carotid cavernous fistula, Tolosa-Hunt syndrome, and meningioma
5.	Orbital syndrome	Commonly seen with proptosis and is frequently accompanied by congestion of conjunctival vessels and conjunctival chemosis
6.	Isolated 6th nerve palsy syndrome	Only lateral rectus weakness and no historical data to implicate a specific pathology
